# Exploring antibiotic knowledge and practices in a sample of damascus residents: a foundation for informed interventions and public health strategies

**DOI:** 10.1186/s12889-026-27053-5

**Published:** 2026-03-21

**Authors:** Abdullah H. Maad, Alissar AlJerf, Atem Bethel Ajong, Muaaz Alajlani, Loai Aljerf

**Affiliations:** 1 Department of Pharmaceutics, College of Pharmacy, University of Al- Ameed, Karbala City, Iraq; 2 Haitham Abdel-Salam School, Ministry of Education, Al-Jama’at District, Jaramanah, Damascus, Syrian Arab Republic; 3 Department of Mother and Child Care, Kekem District Hospital, Kekem, West Region Cameroon; 4 Department of Biochemistry, University of Dschang, Dschang, Kekem, West Region Cameroon; 5 Faculty of Pharmacy, Arab International University, Damascus, Syrian Arab Republic; 6 Key Laboratory of Organic Industries, Department of Chemistry, Faculty of Sciences, Damascus University, Damascus, Syrian Arab Republic

**Keywords:** Antimicrobial resistance, Sociodemographic, Antibiotic use, Public health, Self-medication, Education, Healthcare, Middle East, Reliability, Health behavior

## Abstract

**Background:**

Antimicrobial resistance (AMR) constitutes a formidable global health threat, spanning bacterial, viral, and fungal pathogens. Within this broader context, antibiotic resistance (ABR)—specifically the resistance of bacterial pathogens to antibiotic agents—is the primary focus of this investigation, as it is most directly influenced by community-level knowledge and practices (KP).

**Methods:**

This study employed a cross-sectional survey of 200 participants in Damascus, Syria, to explore the relationships between sociodemographic characteristics and knowledge, attitudes, and practices (KAP) regarding antibiotic use. However, given the cross-sectional nature of the study, the observed relationships are interpreted as associations rather than causal effects.

**Results:**

The findings highlight patterns of antibiotic-related KP among surveyed residents in Damascus and suggest areas where educational and public health initiatives may be beneficial. Our data reveal generally high adherence to prescribed antibiotic courses alongside ongoing behaviors such as self-medication and antibiotic reuse, reflecting persistent gaps in awareness and access barriers. Observed associations between education level, gender, and age with responsible antibiotic behaviors suggest that social determinants may serve as important correlates of health practices within this sample. By exploring community-level factors associated with antibiotic use in a Damascus-based sample, this research provides descriptive data that may help inform the development of local educational initiatives tailored to this specific context.

**Conclusions:**

This exploratory study provides preliminary evidence connecting social and demographic variables to self-reported antibiotic misuse, contributing to the available data regarding antibiotic use behaviors within a specific urban context in Syria and highlighting the utility of behavioral science frameworks as conceptual lenses for future public health interventions to combat AMR effectively.

**Graphical Abstract:**

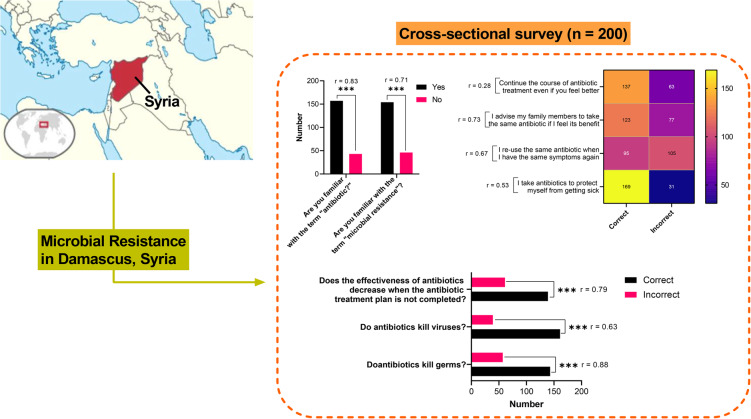

**Supplementary Information:**

The online version contains supplementary material available at 10.1186/s12889-026-27053-5.

## Background

### Educational relevance and implication statement

Optimizing antibiotic use and combating resistance necessitate evidence-based educational initiatives and public health strategies. This study’s findings provide actionable insights for the development of targeted interventions, healthcare provider training programs, and policies that promote responsible antibiotic prescribing practices. By informing educational campaigns and interventions that address knowledge gaps and high-risk behaviors, this research aligns with preserving antibiotic effectiveness and safeguarding public health.

### Introduction

The introduction of antibiotics significantly improved the management of infectious diseases and reduced related mortality [1]. Antimicrobial resistance (AMR) refers broadly to resistance among microorganisms to antimicrobial agents, including antibiotics, antivirals, antifungals, and antiparasitics. Because the present study specifically examines public knowledge and practices (KP) related to antibiotic use, the term antibiotic resistance (ABR) is used throughout this manuscript when referring specifically to resistance to antibacterial drugs. Yet today, AMR is widely recognized as one of the most pressing global public health threats. However, within this broader challenge, ABR—the resistance of bacteria to antibacterial agents—represents a major and rapidly growing component, undermining decades of progress [2]. Misuse and overuse of antibiotics—such as self-medication, incomplete courses, and inappropriate prescribing—accelerate the development of resistant bacterial strains, leading to infections that are harder and more expensive to treat, with higher morbidity and mortality rates [3].

Globally, extensive research has examined public knowledge, attitudes, and practices (KAP) related to antibiotic use, highlighting widespread misconceptions, self-medication behaviors, and gaps in public understanding [4]. Studies across diverse populations have consistently shown that many people incorrectly believe antibiotics are effective for viral infections or fail to recognize the importance of completing prescribed treatments [5]. These knowledge gaps significantly link to misuse and, ultimately, to the acceleration of AMR [6].

In the Middle East, where healthcare access is often strained by political instability and economic hardship, the inappropriate use of antibiotics is especially concerning [7]. In Syria, the protracted conflict and collapse of healthcare infrastructure have exacerbated these challenges [8]. Previous studies conducted in select Syrian regions, such as Kalamoon [9] and during the COVID-19 pandemic [10], have reported alarming rates of non-prescription antibiotic use and poor public understanding of proper antibiotic practices. However, available data remain scarce, fragmented, or outdated, with limited insights into the current situation in the capital, Damascus [11]. This gap is updated, region-specific evidence hinders the ability of public health authorities and policymakers to design targeted, culturally appropriate interventions [12]. To develop effective health education campaigns and antibiotic stewardship strategies, it is crucial to first understand the current state of public knowledge and behaviors [13].

- *Research question 1*: What is the contemporary landscape of antibiotic KAP among Damascus residents, and how do these indicators vary across demographic strata and socioeconomic factors?
- *Research question 2*: To what degree do knowledge gaps and behavioral risks associated with antibiotic use among Damascus residents link to the propagation of AMR, and how can these findings inform the development of culturally sensitive and effective public health strategies?

### Objectives of the study

The study aims to evaluate self-report to antibiotic use based on the exploration of antibiotic-related KP among a sample of residents in Damascus, Syria, and to identify patterns that may inform future research and public health education strategies. Specifically, the primary objectives are to:

- Investigate the current levels of antibiotic literacy and understanding among the study population
- Identify patterns and disparities in antibiotic use and misuse across demographic and socioeconomic strata
- Determine the key factors influencing antibiotic KAP, and their implications for public health interventions

While the study focuses on community-level KAP data, we are situating our findings within the broader, interdisciplinary context of the One Health approach and social-ecological perspectives to discuss the potential systemic implications of antibiotic misuse.

### Research hypotheses

- ▪ *Cognitive-Behavioral Hypothesis*: Deficiencies in antibiotic knowledge among Damascus residents are not isolated but are systematically associated with reported patterns of behavior that correlate with antibiotic misuse.
- ▪ *Sociodemographic Influence Hypothesis*: Variations in antibiotic-related KP are significantly mediated by sociodemographic characteristics—including, but not limited to, age, educational background, income level, and healthcare accessibility—highlighting specific profiles of vulnerability and resilience within the population.
- ▪ *Systemic Association Hypothesis*: Knowledge gaps and self-reported risky antibiotic practices among this sample are potentially associated with broader factors related to local AMR propagation, suggesting community behavior as a relevant area for further investigation.
- ▪ *Intervention-Informing Hypothesis*: By identifying specific knowledge gaps and reported behavioral risks within this sample, these findings can provide a preliminary basis for discussing targeted educational interventions that aim to address local antibiotic practices toward safer and more sustainable patterns.

## Materials and methods

### Study design and setting

This study employed a cross-sectional survey design conducted between March and April 2023 in Damascus, the capital of Syria, targeting the adult resident population. The design aligns with standard practices for KAP research on public health topics [4, 14]. The primary aim was to evaluate contemporary antibiotic-related KAP across demographic and socioeconomic strata, with the broader goal of informing public health strategies to combat antibiotic misuse and AMR. Besides, because the study employed a cross-sectional design, the analyses identify statistical associations rather than causal relationships between variables. Furthermore, as the study is strictly survey-based and did not involve primary microbiological sampling, supplementary contextual information regarding commonly used antibiotics and their typical target pathogens was compiled [15, 16]. This qualitative synthesis is derived from established regional clinical guidelines and existing surveillance data rather than participant laboratory testing. The ABR profiles presented in Tables S2 and S3 of the Supplementary Material (SM) are provided solely to contextualize self-reported behaviors [16, 17]. These data represent a synthesis of local epidemiological reports [18, 19] and are not derived from microbiological sampling of the study cohort. So that, all graphical figures present aggregated results derived from participants’ responses to the structured questionnaire and therefore reflect self-reported knowledge and behavioral practices rather than clinically verified antibiotic usage.

### Target population and sampling strategy

The target population was defined as adults (≥ 18 years) residing in Damascus across all socioeconomic backgrounds. Based on the most recent UN urban population estimates, Damascus hosts approximately 2.5 million residents. To ensure representativeness, we employed stratified random sampling across major districts (e.g., Al-Midan, Al-Muhajirin, Al-Mazzeh, Rukn al-Din, and Barzeh), capturing variability by age, gender, education, income, and healthcare access. To ensure methodological validity, the study applied a stratified random sampling strategy, minimized selection bias through proportional recruitment across districts, and used trained interviewers to reduce interviewer and social desirability biases.

#### Sample size determination

Sample size was calculated using the Raosoft online calculator (http://www.raosoft.com/samplesize.html), inputting a population size of 2.5 million, 95% confidence interval (CI), 5% margin of error, and an assumed 50% response distribution (due to no prior prevalence data on KAP in this population). The minimum required sample size was calculated to be 384 participants based on standard population proportion assumptions; however, due to logistical and resource constraints during the study period, the final number of completed questionnaires included in the analysis was 200 participants, a number comparable to similar KAP studies in low-resource urban settings (e.g. [2, 9, 13, 20–24]; associated range of sample sizes (n) = 178–434), acknowledging this as a study limitation.

Consequently, the study sample consisted of 200 participants, which is below the originally estimated target size and therefore limits statistical power and generalizability. The study is subject to selection bias stemming from our recruitment strategy. Because data were collected via face-to-face interviews in public spaces, the sample is disproportionately composed of young females (aged 18–30). This demographic cluster likely over-represents individuals with higher accessibility to public social spaces and may not capture the antibiotic practices of more reclusive, elderly, or male-dominated workforce segments. Consequently, the findings should be interpreted as an exploratory snapshot of this specific urban subgroup rather than a comprehensive demographic profile of the city. Given the sample size and recruitment strategy, these findings are considered exploratory; while they offer useful local insights, they are not generalizable to the broader Syrian population or the entire Damascus municipality.

### Questionnaire development and validation

The survey instrument was developed following established guidelines on survey design and item generation for KAP studies [25, 26]. We systematically reviewed validated KAP questionnaires from related AMR research [24, 27] to adapt content relevant to the Syrian context. The final instrument comprised three sections:

- Section 1: Sociodemographic Characteristics — 7 items (age, gender, marital status, education level, household income, employment status, and health insurance coverage). Differences in knowledge scores were observed across categories of employment status.
- Section 2: Antibiotic Knowledge — 5 statements assessing awareness of antibiotic indications, resistance, importance of treatment adherence, and familiarity with specific antibiotics (Azithromycin, Augmentin, Amoxicillin, Levofloxacin, and Ciprofloxacin).
- Section 3: Practices and Attitudes — 9 items covering sources of antibiotics, reasons for use, adherence behavior, self-medication, and sharing practices.

The questionnaire was translated into Arabic and underwent forward and backward translation to ensure linguistic accuracy [28]. A pilot test was conducted with 30 participants’ representative of the target population. Based on feedback, minor adjustments were made for clarity and cultural appropriateness.

To assess reliability, we calculated Cronbach’s alpha for the KP sections, yielding values of 0.78 and 0.81 respectively, indicating acceptable internal consistency [27]. Content validity was further ensured through expert review by two public health specialists and one pharmacologist. The full survey instrument is provided in the SM file to support replicability. Detailed protocols for sampling, data collection, and analysis are described herein to enable reproducibility by other researchers.

### Data collection procedure

Trained surveyors conducted face-to-face structured interviews in public spaces (parks, markets, and community centers) to accommodate varying literacy levels. While this approach facilitated engagement with a diverse range of residents, we acknowledge that recruitment in public areas during daytime hours may have introduced selection bias, potentially favoring individuals who frequent these locations, such as students or those with flexible schedules. Participation was voluntary, and all participants provided written informed consent after the study’s purpose, confidentiality, and data handling procedures were explained. To enhance response quality, surveyors received standardized training on ethical conduct, interview techniques, and data recording protocols.

### Methodological scope and theoretical contextualization

It is essential to clarify that this investigation is solely survey-based and did not involve primary microbiological sampling or laboratory testing. All references to AMR patterns—including resistance rates for *Escherichia coli*, *Klebsiella pneumonia*, and *Staphylococcus aureus*—are sourced from existing local surveillance data. Additionally, to contextualize the observed patterns of antibiotic knowledge and self-reported practices, it is useful to consider relevant behavioral and public health frameworks that have been applied in previous research on antibiotic use [15, 29, 30].

Models such as the Health Belief Model (HBM), the Theory of Planned Behavior (TPB), and the Capability, Opportunity, Motivation, and Behavior (COM-B) framework have been widely used to interpret determinants of health-related behaviors. However, it is important to emphasize that the present study did not directly measure the theoretical constructs associated with these models (e.g., perceived susceptibility, subjective norms, perceived behavioral control, or motivation). Therefore, these frameworks are referenced here solely as conceptual lenses for interpretation rather than as empirically tested models within this study.

### Study variables

- Outcome variables
  - ▪ Knowledge score (based on correct responses to knowledge items).
  - ▪ Practice score (based on adherence and appropriate use patterns).

- Independent variables
  - ◦ Sociodemographic variables (age, gender, educational level, monthly income, marital status, occupational status, and insurance coverage). In this study, the term “employment status” refers to participants' current occupational situation (e.g., student, employed, self-employed, unemployed, homemaker, or retired), rather than years of professional experience.
  - ◦ Specific antibiotic use familiarity (named antimicrobials).

All demographic descriptors were defined according to standard population survey categories to ensure clarity and comparability with previous public health studies. The variables were carefully selected to align with the study’s research questions and hypotheses on cognitive-behavioral patterns, sociodemographic influences, and systemic impacts on AMR.

### Statistical analysis

All data were analyzed using IBM SPSS Statistics version 28 (IBM Corp., Armonk, NY). Descriptive statistics summarized frequencies, means, and standard deviations.

For inferential analyses:

- Associations between categorical variables (e.g., knowledge level vs. education) were tested using Chi-square tests.
- Associations involving ordinal or continuous outcomes (e.g., knowledge score vs. income) were assessed using Mann–Whitney U tests (for two-group comparisons) or Kruskal–Wallis tests (for multi-group comparisons) (Table S1), given the nonparametric nature of KAP scores.
- Multivariable logistic regression (Table S1) was conducted to identify independent associations with good knowledge and good practice, adjusting for covariates.
- A detailed correlation matrix featuring all study variables, including demographic factors and specific behavioral items, is provided in Table S7 to provide a comprehensive overview of the interrelationships without overcomplicating the main text.

All tests were two-tailed, with significance set at *p* < 0.05.

Additionally, although correlation and regression analyses are conducted to explore relationships between variables, the cross-sectional nature of the study precludes inference regarding directionality or prediction. The statistical models therefore identify associations between antibiotic knowledge, sociodemographic characteristics, and self-reported behaviors, but they do not establish causal or predictive relationships. So, the statistical analyses identify associations between variables rather than directional or predictive relationships, and the results are therefore interpreted accordingly.

### Data visualization

Graphs and visual summaries were generated using GraphPad Prism version 8 (GraphPad Software, La Jolla, CA), a widely used platform for producing publication-quality visualizations.

### Ethical considerations

This study adhered to the ethical principles outlined in the Declaration of Helsinki [31]. Ethical approval was obtained from the Institutional Review Board of Damascus University (DU), reference number [DU-FSDC-IRB-1097]. Participants provided informed consent prior to participation, and all data were anonymized and securely stored. No financial or non-financial conflicts of interest were identified.

## Results

### Demographic characteristics of survey participants

The survey achieved a high response rate of 89% (200 out of 225), reflecting strong participant engagement (Fig. S1). The sample represented a range of educational levels and occupational categories, including students (35%, *n* = 70), individuals employed in the education sector (25%, *n* = 50), and other technical or service-based occupations (40%, *n* = 80). Regarding length of current employment or residence, 55% (*n* = 110) reported a duration of over five years, 30% (*n* = 60) reported 2–5 years, and 15% (*n* = 30) reported less than two years.

#### Demographic profile and association with antibiotic knowledge

**Fig. 1:**
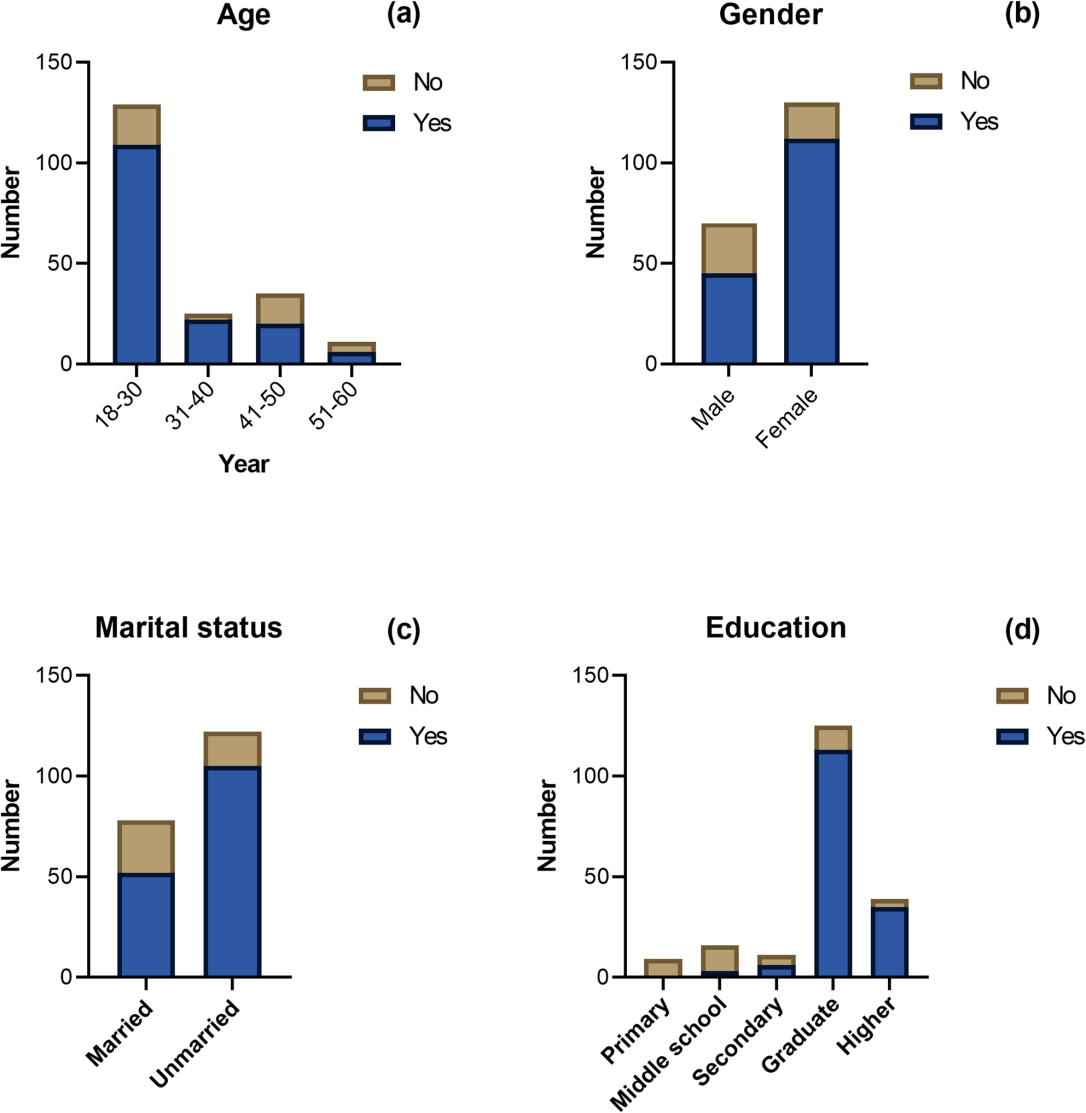
Association between participants’ sociodemographic characteristics and survey-measured antibiotic knowledge scores among respondents in Damascus (*n* = 200). Panels show relationships between knowledge levels and (**a**) age (*p* = 0.001), (**b**) gender (*p* = 0.0001), (**c**) marital status (*p* = 0.001), and (**d**) educational attainment (*p* = 0.0001). Knowledge data represent responses to structured questionnaire items assessing awareness of antibiotic use and resistance

The survey also captured data across age, gender, and geographical location (Fig. 1). Most participants were females aged 18–30. This demographic skew suggests that our sample primarily reflects the perspectives of younger, female residents, which may limit the representativeness of the findings regarding older populations or male residents in Damascus. Geographically, respondents were distributed across urban (40%, *n* = 80), suburban (30%, *n* = 60), and rural areas (30%, *n* = 60). These diverse demographics provided a well-rounded perspective, supporting the educational aims of the study.

### Knowledge and awareness of antibiotic use

**Fig. 2:**
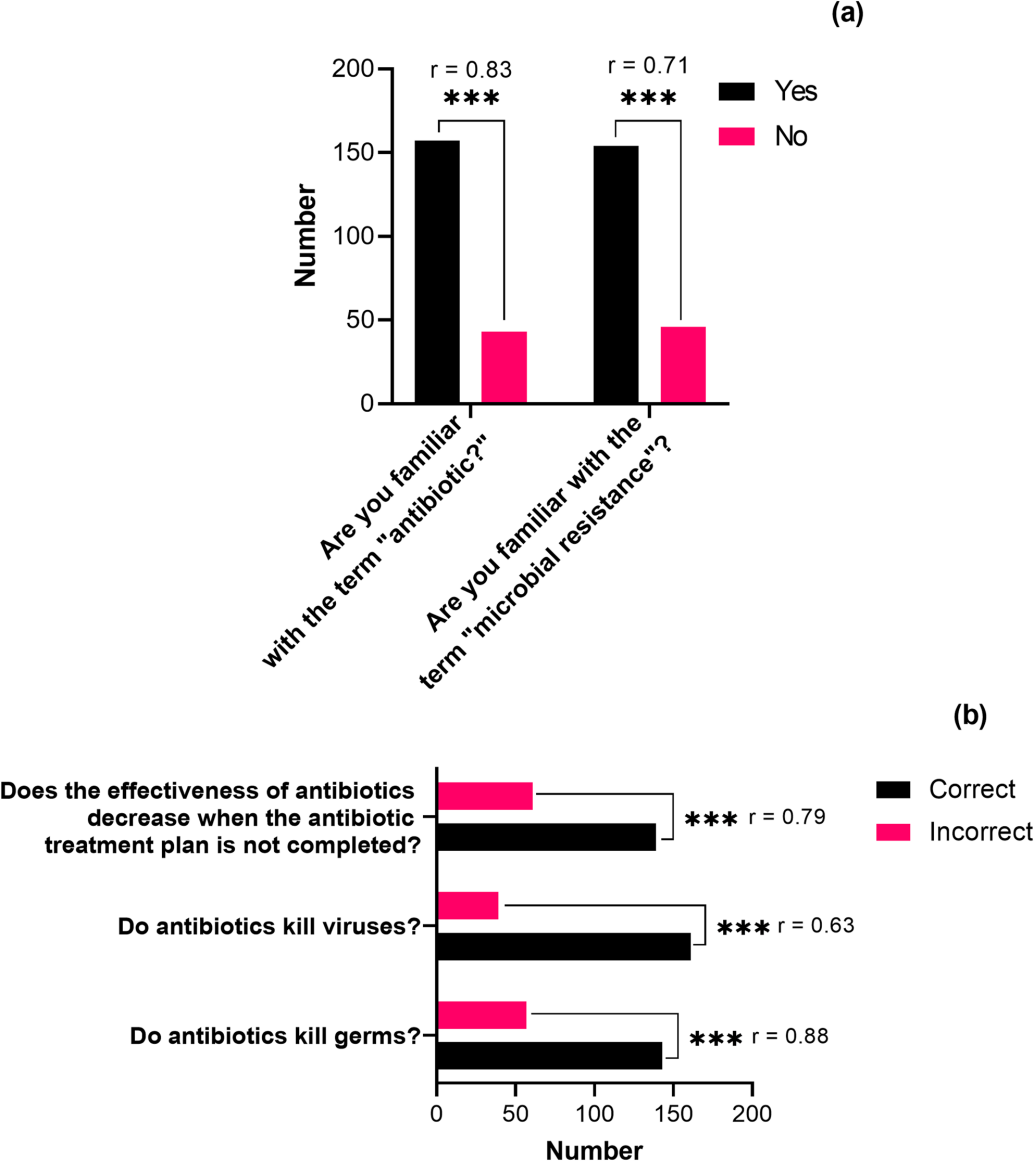
Distribution of participants’ self-reported knowledge regarding antibiotic use and antibiotic resistance among surveyed residents of Damascus (*n* = 200). Responses were derived from structured questionnaire items and categorized as (**a**) Yes/No awareness responses and (**b**) Correct/Incorrect knowledge responses

Among the 200 respondents, 79% were familiar with the term *antibiotic*, and 77% knew about AMR (Fig. 2). This reflects a solid foundation of awareness in this sample of residents in Damascus, although additional education is necessary to ensure that this knowledge translates into proper practices. Notably, 61% of participants reported prior antibiotic use, with 45% admitting to self-medicating without consulting healthcare professionals—a concerning trend. Moreover, 52% did not know how to properly dispose of unused antibiotics, which raises environmental and resistance concerns.

To contextualize the public health significance of our findings, we refer to the general efficacy profiles of commonly used antibiotics in the city (see Supplementary Table S3). The relative resistance rates (e.g., for *E. coli* and *K. pneumonia*) are derived from urban surveillance data rather than primary sampling from the 200 participants. These resistance data serve as an external benchmark rather than a direct microbiological analysis of the study cohort.

Antibiotics, such as penicillin, amoxicillin, ciprofloxacin, and doxycycline, are critical in treating bacterial infections like *S. aureus*, *E. coli*, and *K. pneumonia*, but they are ineffective against viral infections like the flu or the common cold. Alarmingly, many respondents believed it was acceptable to stop antibiotics once symptoms improve, despite the known risk of contributing to ABR if the full course is not completed. These insights highlight the need for expanded public health campaigns focusing on appropriate antibiotic use and the risks of misuse.

#### Antibiotic use patterns, knowledge gaps, and educational implications

**Fig. 3:**
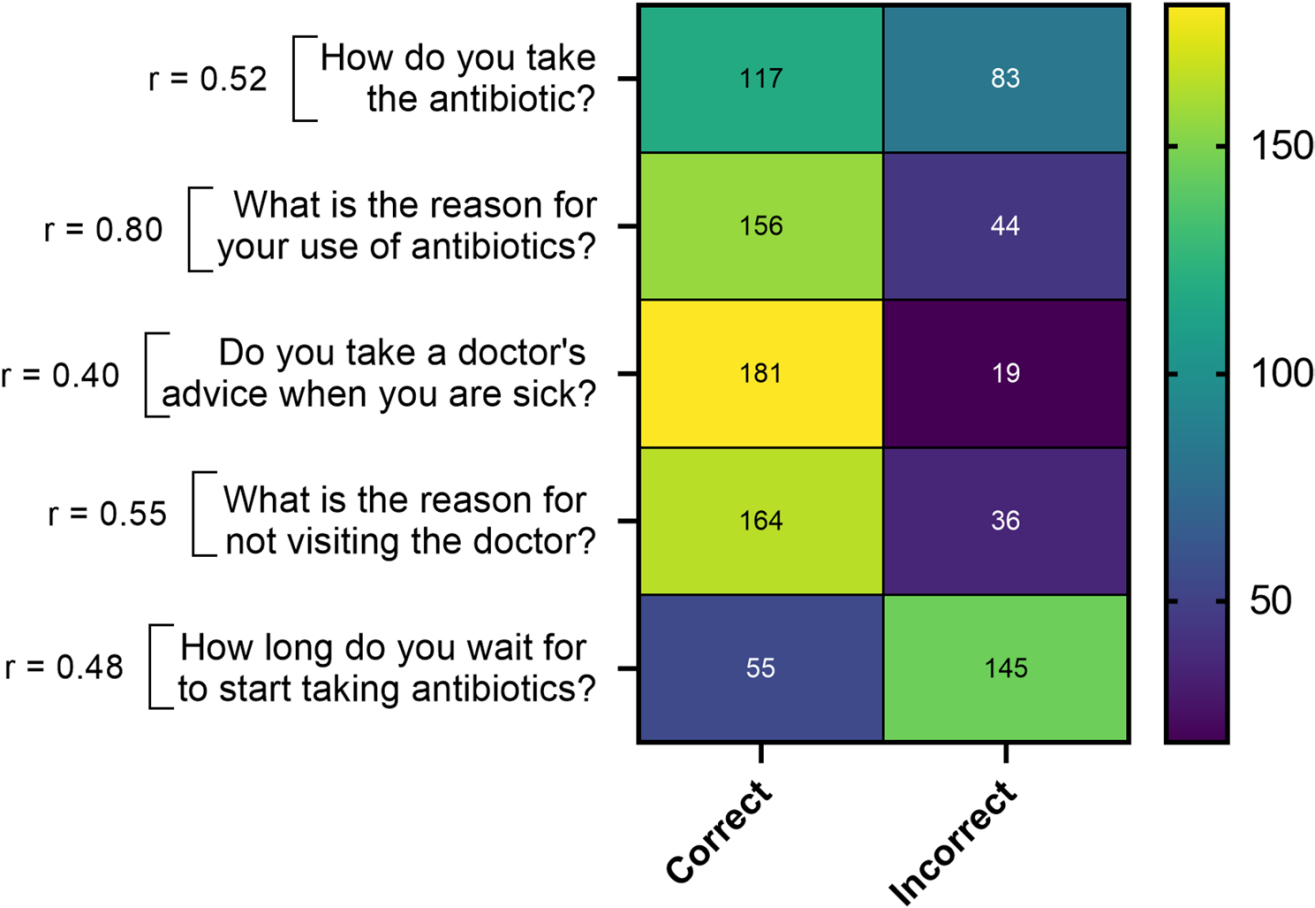
Self-reported antibiotic use practices among surveyed residents of Damascus (*n* = 200), based on questionnaire responses describing behaviors such as consultation with healthcare professionals, treatment adherence, and antibiotic acquisition patterns (*p* = 0.0001)

The analysis of survey data (*n* = 200) revealed important insights into antibiotic use patterns, behavioral drivers, and knowledge gaps among the sample population. As shown in Table 1; Fig. 3, while over 59% of participants correctly answered questions on appropriate antibiotic use, consulting a doctor, and reasons for seeking medical advice, a substantial 72% provided incorrect responses regarding the optimal timing for initiating antibiotics. This points to a significant educational gap that requires targeted intervention to mitigate misuse and curb AMR.

**Table 1:** Distribution of antibiotic use behaviors and awareness (*n* = 200)

Behavioral/Awareness Category	Frequency (*n*)	Percentage (%)	*p*-value*
Responsible Practices			
Adherence to doctor’s instructions	181	90.5	< 0.001
Completing the full antibiotic course	131	65.5	0.002
Consulting a doctor before use	117	58.5	0.005
Risk Behaviors			
Using leftover antibiotics	84	42.1	0.001
Sharing antibiotics with family/friends	71	35.5	0.003
Knowledge & Awareness			
Awareness of Antibiotic Resistance (ABR)	162	81.2	< 0.001
Knowledge of ABR Mechanisms	126	63.2	0.004

*Statistically significant at *p* < 0.05 (Chi-square test)

A detailed breakdown of behavioral patterns indicated that 81.2% of respondents were aware of ABR, and 90.5% reported adherence to doctors’ instructions, with an average antibiotic course lasting 7.4 days. Knowledge of ABR mechanisms was present in 63.2% of participants. Encouragingly, 65.5% completed their full antibiotic course and 58.5% sought medical consultation prior to use — both critical elements of responsible antibiotic behavior. However, concerning trends emerged: 42.1% admitted to using leftover antibiotics from prior prescriptions, and 35.4% engaged in sharing antibiotics with family or friends, practices known to elevate the risk of resistance development.

Importantly, all variables related to antibiotic use practices were statistically significant (*p* < 0.05), underscoring the observed associations (see the SM). The results highlight not only the population’s partial understanding of antibiotic use but also the behavioral patterns that undermine effective antibiotic stewardship. Educational initiatives should therefore prioritize correcting misconceptions about antibiotic initiation timing, reducing self-medication and sharing practices, and reinforcing the importance of completing prescribed courses. These insights provide a critical evidence base for designing public health strategies to promote responsible antibiotic use and reduce the growing threat of AMR in Syria.

#### Antibiotic use behavior and awareness

**Fig. 4:**
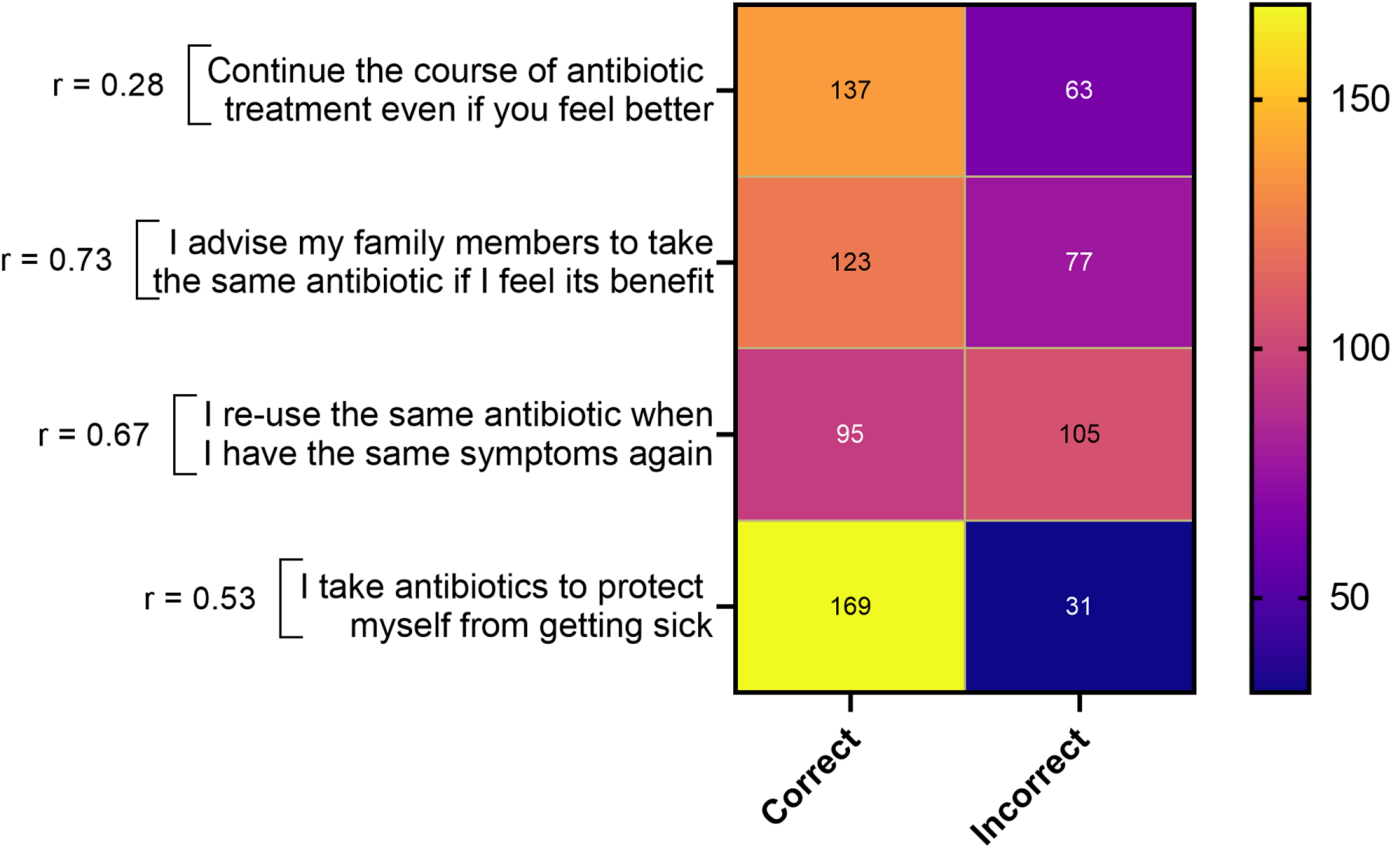
Self-reported behavioral patterns related to antibiotic use among surveyed residents of Damascus (*n* = 200), including reported adherence to prescribed treatments, reuse of leftover antibiotics, and consultation practices with healthcare professionals (*p* = 0.0001)

Promisingly, over 60% of respondents reported completing their antibiotic courses even when feeling better, avoiding antibiotics solely for prevention, and appropriately advising family members (Fig. 4). However, 52% admitted to reusing old antibiotics for similar symptoms without medical consultation—an unsafe practice that increases resistance risks (Fig. 4).

Understanding antibiotic specificity is crucial; for example, β-lactam antibiotics target Gram-positive bacteria, while macrolides are more effective against Gram-negative pathogens. Misusing or reusing antibiotics without professional input can disrupt the body’s microbiome and fuel resistance.

The statistically significant correlations (*p* < 0.05) between behavioral questions and responses underscore the need for sustained educational campaigns and policy interventions to reinforce proper antibiotic use.

#### Correlation analysis of antibiotic knowledge and behavior

Correlation analysis (Table 2) and the broader correlation matrix provided in the Table S7 indicates to the significant associations between participants’ antibiotic knowledge levels and their subsequent reported practices. Overall, a moderate-to-strong positive correlation was observed between the composite Knowledge Score and the composite Practice Score (*r* = 0.51, *p* < 0.01). Detailed item-level analysis further illustrates that specific areas of knowledge are strongly associated with responsible behavior. For instance, awareness of antibiotic stewardship is strongly correlated with the likelihood of reporting adverse side effects to a healthcare professional (*r* = 0.75, *p* < 0.01). Similarly, a strong positive association was found between the understanding of antibiotic indications and the propensity to consult a physician prior to initiating treatment (*r* = 0.65, *p* < 0.001). Conversely, knowledge gaps regarding antibiotic classification and dosage instructions were inversely associated with risky behavioral patterns. A significant negative correlation was identified between knowledge of antibiotic classification and the frequency of self-medication (*r* = -0.50, *p* = 0.010), as well as between understanding dosage requirements and the tendency to share leftover antibiotics with peers (*r* = -0.45, *p* = 0.015).

**Table 2:** Associated factors and correlations of antibiotic knowledge and practice (*n* = 200)

Knowledge Variable (Independent)	Behavioral Practice (Outcome)	Pearson’s *r*	Adjusted Odds Ratio (OR)	95% Confidence Interval (CI)	*p*-value
Promoting Responsible Use					
Overall Knowledge Score	Practice Score (Composite)	0.51	—	—	< 0.01
* Antibiotic definition*	Completing full antibiotic course	0.70	2.5	1.8–3.5	< 0.001
Importance of stewardship	Reporting side effects to a doctor	0.75	3.5	2.5–4.9	< 0.001
* Knowledge of indications*	Consulting a doctor before use	0.65	2.2	1.5–3.2	< 0.001
Resistance awareness	Avoiding antibiotic misuse	0.60	1.8	1.2–2.7	0.002
* Role of pharmacists*	Seeking advice on antibiotic use	0.70	3.0	2.0–4.2	< 0.001
Identifying Risk Factors					
Knowledge of classification	Self-medication	-0.50	0.6	0.4–0.9	0.010
* Dosage instructions*	Sharing antibiotics	-0.45	0.5	0.3–0.8	0.015
Medication interactions	Use for viral infections	-0.40	0.4	0.2–0.7	0.020

OR > 1 indicates a higher likelihood of positive behavior with increased knowledge; OR < 1 indicates a lower likelihood of risky behavior as knowledge increases. All values are derived from the primary analysis and Table S7

Multivariable logistic regression analysis, summarized in Table 2, confirmed these bivariate findings. After adjusting for potential confounders, higher antibiotic literacy remained strongly associated with responsible use; specifically, a one-unit increase in the knowledge score significantly increased the likelihood of an individual completing their prescribed antibiotic course (OR: 2.5, 95% CI: 1.8–3.5, *p* < 0.001). These findings support our cognitive-behavioral hypothesis, suggesting that targeted interventions aimed at closing specific knowledge gaps could effectively reduce high-risk antibiotic misuse in this population.

### Antibiotic usage, spectrum, and resistance profiles

In the present study, pathogen–antibiotic relationships are discussed only as contextual background to interpret antibiotic use behaviors reported by survey participants. Analysis of antibiotic usage patterns and microbial susceptibility revealed several notable trends (Tables 3 and 4, S2, and S3). Among the surveyed population, Amoxicillin was the most commonly used antibiotic (38.2%), followed by Augmentin (26.7%), Ciprofloxacin (18.4%), Levofloxacin (9.5%), and Azithromycin (7.2%) (Table S3). These antibiotics varied in their spectrum of activity and targeted bacterial pathogens. For instance, β-lactam antibiotics such as Amoxicillin primarily targeted *S. pneumonia* and other Gram-positive bacteria, while macrolides like Azithromycin were more effective against Gram-negative organisms such as *Haemophilus influenzae* (Table S2).

**Table 3:** Commonly used antibiotics, target bacteria, and spectrum of activity

Antibiotic	Class	Common Target Bacteria	Spectrum of Activity	Clinical Usage (%)
Amoxicillin	β-lactam (Penicillin)	*S. pneumonia*, *E. coli*	Primarily Gram-positive	38.2%
Augmentin	β-lactam + β-lactamase inhibitor	*S. aureus*, *H. influenza*	Broad-spectrum	26.7%
Ciprofloxacin	Fluoroquinolone	*E. coli*, *K. pneumonia*	Broad-spectrum	18.4%
Levofloxacin	Fluoroquinolone	*S. pneumonia*, *Pseudomonas*	Broad-spectrum	9.5%
Azithromycin	Macrolide	*H. influenza*, *M. catarrhalis*	Primarily Gram-negative	7.2%

**Table 4:** Resistance rates of key pathogens to selected antibiotics (%)

Bacterial Species	Amoxicillin	Ciprofloxacin	Azithromycin	Levofloxacin
*E. coli*	43%	28%	15%	24%
*S. aureus*	31%	22%	19%	27%
*K. pneumonia*	55%	34%	12%	30%
*S. pneumonia*	38%	16%	21%	18%

Antibiotic efficacy profiles suggested that Ciprofloxacin had consistently high activity against *E. coli* and *K. pneumonia*, whereas Amoxicillin was most effective against *S. pneumonia* (Table S3). Resistance data further may indicate these trends: resistance to Amoxicillin was notably high in *K. pneumonia* (55%) and *E. coli* (43%), while resistance to Ciprofloxacin and Levofloxacin was comparatively lower across most species, ranging from 16% to 34% (Table 4).

## Discussion

**Fig. 5:**
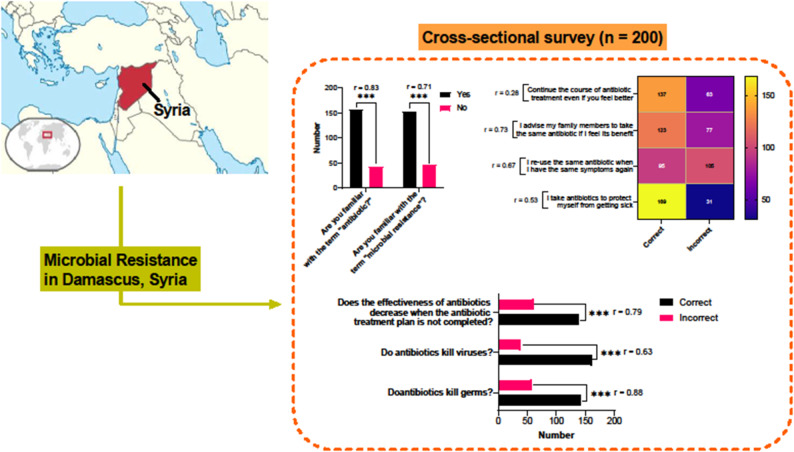
Damascus, Syria survey reveals associations between resident knowledge and practices on antibiotic resistance

This study provides exploratory insights into patterns of antibiotic knowledge and self-reported practices among residents of Damascus (Fig. 5), highlighting associations between sociodemographic characteristics and responsible antibiotic use (Fig. S2). While educational attainment remains the strongest correlate of antibiotic literacy, the high rate of self-medication (52%) suggests that knowledge is frequently superseded by economic necessity and cultural norms.

### Education as a cornerstone of antibiotic literacy and behavior

Our results identified a statistically significant association between higher educational levels and better antibiotic knowledge scores (Tables S4, S5, and S6, and Fig. 1; *p* < 0.001), which aligns with existing literature highlighting education as a primary determinant of health literacy [13, 21]. This association likely reflects the role of education in strengthening health literacy and the ability to interpret medical guidance regarding antibiotic use. Statistically, participants with tertiary education were 2.5 times more likely to indicate responsible antibiotic behaviors such as completing full courses (Table 2, OR 2.5), suggesting that higher educational attainment may be associated with more responsible antibiotic use behaviors. These results are consistent with behavioral research suggesting that increased knowledge is often associated with improved health practices [32]. Conversely, the lack of significant association between knowledge and other sociodemographic variables such as age and income (*p* > 0.05) suggests that education appears to be an important factor associated with improved antibiotic literacy, perhaps due to the relatively homogeneous low-income status of the sample (80% reporting poor financial conditions) (Fig. S1), which may have diminished income’s discriminatory power (Table S7).

### Interdisciplinary insights: integrating social sciences, economics, and humanities

The findings of this study extend beyond biomedical dimensions, revealing rich interdisciplinary implications that touch the social, economic, and ethical layers of public health (Table S8). The observation that a majority of respondents reported adherence to prescriptions and completion of antibiotic courses (as shown in Table 2) suggests not only a positive individual knowledge base but also underlying social dynamics such as trust in medical authority, family health norms, and peer influence. These patterns echo constructs from the social sciences, particularly the TPB (Table S9), which emphasizes how attitudes, subjective norms, and perceived behavioral control are associated with health behaviors [33]. In the context of Damascus, a city marked by social resilience and cultural complexity, these findings underscore the importance of designing public health campaigns that are not only factually accurate but also culturally tailored and socially resonant [34].

From an economic standpoint, the study’s results point to the substantial downstream costs associated with antibiotic misuse. While nearly 70% of respondents displayed good practices, the remaining proportion exhibiting gaps or risky behaviors signals a population-level economic risk: the potential for increased healthcare expenditures, the need for costlier second-line drugs, and losses in labor productivity (Table S10). These data resonate with global estimates indicating that AMR could cost the global economy up to $100 trillion by 2050 if left unaddressed [35]. This aligns with World Bank projections, which warn that AMR could push up to 28 million people into extreme poverty by 2050, disproportionately impacting low- and middle-income countries [36, 37]. Therefore, the call for targeted, preventive public health interventions (Table S11) is not just a clinical necessity but an economic imperative, aligning local action with international health financing priorities and cost-containment strategies.

The humanities lens adds further depth to the interpretation of these results. The identified gaps in antibiotic knowledge and the persistence of misconceptions (Table 2) raise ethical and cultural questions: *How do historical experiences*,* such as conflict*,* healthcare access limitations*,* or collective trauma*,* influence present-day health decision-making? What responsibilities do individuals bear toward community health*,* especially in settings where healthcare resources are stretched?* The ethical framework of antibiotic stewardship becomes particularly salient here, reframing antibiotic misuse not merely as a personal lapse but as a breach of intergenerational responsibility and social solidarity [38]. Moreover, cultural narratives around medicine, illness, and trust in biomedical authority — often shaped by historical legacies and societal disruptions — can powerfully link to antibiotic use patterns, requiring that interventions engage not only individuals but also collective cultural scripts [39].

Together, these perspectives highlight that antibiotic use behaviors are embedded within broader social, economic, and cultural contexts. These exploratory findings (Table S11) suggest that interdisciplinary perspectives could be valuable in addressing ABR, potentially informing the development of context-sensitive interventions.

### Persistent unsafe practices amid adequate knowledge

Despite encouraging knowledge levels, our data revealed that 52% of respondents admitted to reusing leftover antibiotics without consulting healthcare professionals (Fig. 4; Table S12). This dissonance between knowledge and practice (Table S7) is justified by entrenched socioeconomic and cultural factors. Financial constraints and limited health insurance coverage (61% uninsured) likely contribute to reliance on self-medication and reduced consultation with healthcare providers [40–42]. Moreover, cultural perceptions that regard antibiotics as readily accessible “cure-alls” perpetuate self-medication, reinforced by social norms and anecdotal experiences [43]. These findings suggest that improving antibiotic knowledge alone may not be sufficient to ensure responsible practices. Similar patterns of self-medication have been reported in other Middle Eastern populations [44, 45]. These parallels suggest that despite varying levels of health literacy, the practice of obtaining antibiotics without a prescription is a deeply rooted behavioral norm in Middle Eastern healthcare landscapes [46].

### Role of pharmacists and healthcare professionals

Pharmacists were found to be pivotal influencers (Tables S5 and S8) in shaping antibiotic use, with strong correlations between pharmacist engagement and responsible behaviors (*r* = 0.70, *p* < 0.01; Table 2). Our findings align with global evidence [47, 48] positioning pharmacists as the primary community touchpoint for antibiotic stewardship. Given that 41.3% of our sample relies on pharmacists for information, empowering this sector through standardized counseling protocols is a critical alternative to physician-led interventions in resource-constrained settings. This association likely reflects the accessibility of community pharmacists as primary points of healthcare contact (Fig. S2). Hence, empowering pharmacists via training and policy frameworks could significantly mitigate misuse (Table S7), especially in settings like Damascus where informal antibiotic dispensing is prevalent. Our findings further support an association between antibiotic knowledge and stewardship practices [48, 49], though the cross-sectional nature of this study precludes definitive causal inference.

### Environmental and agricultural considerations

Our study uncovered limited awareness of the contribution of antibiotic use in agriculture to AMR (Table 2), suggesting an important knowledge gap. The identified knowledge gap regarding agricultural AMR drivers highlights the necessity of adopting a One Health framework in future local policy, as detailed in our recommendations [50]. The present study does not directly examine these broader dimensions but highlights behavioral factors that may be relevant to this larger framework. This limited awareness may reflect the indirect relationship between agricultural practices and human health, compounded by scarce public discourse on this topic in Syria. Given that 60% of local farmers reportedly use antibiotics in livestock (Results section), this sector may represent a potential contributor to AMR dynamics, although this study did not directly measure microbiological resistance, necessitating targeted educational and regulatory efforts integrating environmental and agricultural stakeholders [51].

### Demographic disparities in knowledge and behavior

Statistical analyses revealed significant disparities in antibiotic knowledge across age, gender, marital status, and education (*p* < 0.05), reflecting the multifaceted influence of social factors associated with health behavior patterns (Fig. 1; Table S7).

Younger, female, and unmarried individuals exhibited distinct knowledge and behavior profiles, possibly influenced by differential health information exposure, caregiving roles, and social responsibilities [52]. These differences suggest the need for targeted public health interventions, enhancing relevance and efficacy. For instance, targeting younger adults and females with culturally appropriate messaging could leverage their higher receptivity and influence within families.

### Predictive value of knowledge for responsible behavior

The multivariable analysis (Table 2) identified that higher antibiotic knowledge scores were independently associated with more responsible reported use behaviors within the study population. These results further support an association between higher knowledge scores and more responsible antibiotic use (Table S12) [53]. The relatively moderate effect size suggests that while improving knowledge is critical, supplementary factors such as attitudes, social norms, and healthcare system facilitators must also be engaged to optimize outcomes (Tables S7 and S11).

### Bridging knowledge and behavior: a call for integrated strategies

In light of these findings, addressing the antibiotic misuse challenge in Damascus necessitates a multipronged strategy. Educational initiatives should prioritize groups with lower educational attainment and address misconceptions regarding appropriate antibiotic use (Table S13). Simultaneously, structural barriers such as healthcare affordability and access (Table S11) must be mitigated to translate knowledge into practice effectively. Furthermore, the strong pharmacist-patient dynamic calls for policies reinforcing pharmacists’ stewardship roles [22, 47, 54]. Public health messaging may benefit from acknowledging broader environmental and agricultural dimensions of AMR [55, 56]. Finally, culturally ingrained behaviors such as antibiotic sharing and self-medication (Tables S6 and S11) require innovative, community-engaged approaches leveraging behavioral science principles, peer influence, and continuous public dialogue to shift social norms meaningfully [57].

### Implications of usage patterns and emerging resistance

While research on antibiotic KAP has expanded across the Middle East—including comprehensive studies in Lebanon [58], the UAE [44], and multi-country assessments involving seven regional nations [46]—there remains a critical data gap within the Syrian population. This study seeks to build upon these civic insights by providing a localized analysis of Damascus residents. Within the Syrian context, the AMR data referenced were not generated from primary microbiological analyses within the current sample. Instead, these data were drawn from an urban surveillance we performed to provide contextual background regarding the broader AMR landscape. The observed patterns in antibiotic use reported by participants highlight potential concerns related to antibiotic stewardship. The high reliance on Amoxicillin and Augmentin—despite escalating resistance rates in pathogens like *K. pneumonia* and *E. coli*—suggests potential overuse or empirical prescribing not aligned with susceptibility profiles (Table S4) [59, 60]. In particular, the elevated resistance of *K. pneumonia* to Amoxicillin (55%) and Levofloxacin (30%) (Table 4) raises concern over treatment efficacy in common infections such as urinary and respiratory tract infections. The misconception that antibiotics are effective against viral infections remains a persistent challenge across the region. Our findings align with data from Lebanese university students [58] and pilgrims attending the Hajj [61], where high rates of misunderstanding regarding antibiotic indications were also observed. This suggests that the ‘viral-antibiotic’ knowledge gap is a cross-border public health issue requiring regional educational strategies [46].

Furthermore, the data support a misalignment between clinical usage rates and antibiotic performance. For example, Ciprofloxacin, which showed the highest efficacy against multiple Gram-negative pathogens, was used in less than one-fifth of cases (18.4%) (Table 3), despite its demonstrated lower resistance rates in key pathogens. This may reflect prescribing inertia, accessibility issues, or lack of updated resistance guidance in clinical settings. These observations suggest the importance for context-specific antibiotic stewardship strategies and continued surveillance of local resistance data.

Tailored interventions should prioritize shifting use toward more effective, lower-resistance options and away from agents like Amoxicillin where resistance is high. Additionally, the integration of pathogen-specific resistance surveillance into public health strategies could improve empirical therapy and slow the development of ABR.

### Theoretical implication

The findings can be interpreted within established health behavior framework, thereby enriching their explanatory power in the context of AMR and stewardship. While our data do not allow for the formal testing of behavioral models, frameworks such as COM-B provide a useful structure for interpreting how the identified knowledge gaps (Capability) and social determinants (Opportunity) might relate to reported antibiotic practices (Behavior).

Differences observed across employment categories may reflect variations in access to health information and exposure to health-related knowledge in different occupational contexts. Some of the observed patterns may be conceptually consistent with elements described in the HBM, particularly regarding awareness of risks associated with inappropriate antibiotic use. However, because the present survey did not directly assess HBM constructs such as perceived susceptibility, perceived severity, or perceived barriers, these connections should be interpreted cautiously and viewed as illustrative rather than confirmatory (Table S14) [62]. The significant correlations between knowledge variables—such as ABR awareness and side effect recognition—and positive antibiotic use behaviors (Table 2; Fig. 4) are conceptually consistent with the roles of perceived threats and benefits, as described in health behavior frameworks. This supports the theoretical premise that enhancing individuals’ knowledge about the risks of misuse and resistance (Table S4) can increase their likelihood of engaging in protective behaviors like completing full antibiotic courses and consulting healthcare providers.

Furthermore, the influence of demographic factors (e.g., age and education level) and social mediators (pharmacist advice) identified in this research complements the TPB (Table S9) [33], which highlights attitudes, subjective norms, and perceived behavioral control as factors of intention and behavior. The differential knowledge levels across demographic groups and the critical role of pharmacists suggest that subjective norms and social pressures within a community can either facilitate or hinder responsible antibiotic practices. This finding extends TPB (Table S9) by emphasizing the contextual and community-based determinants of antibiotic stewardship (Table S11), echoing calls for integrating social influences into health behavior models [63].

While the study did not directly measure all constructs of the COM-B model, our findings can be interpreted through this lens to hypothesize potential behavioral drivers (Table S15) [64]. Within the COM-B framework, behavior reflects interactions between capability, opportunity, and motivation. While knowledge (capability) was relatively high, external factors such as limited access to healthcare (opportunity) and ingrained cultural habits (motivation) likely undermine behavior change. This suggests that comprehensive theoretical models of antibiotic use must incorporate systemic and environmental constraints alongside individual cognitive factors to fully capture the determinants of misuse.

The incorporation of agricultural antibiotic use awareness into the participants’ knowledge also aligns with the One Health framework, which integrates human, animal, and environmental health domains [65]. This broader ecological perspective expands traditional health behavior theories by recognizing the interconnectedness of AMR drivers beyond individual-level factors, reinforcing the need for multidisciplinary and multisectoral theoretical models.

In summary, behavioral frameworks such as the COM-B model and other health behavior theories have been used in previous research to interpret antibiotic-related behaviors [66–68]. While the present work did not directly measure constructs from these frameworks, the observed patterns of knowledge and reported practices may be conceptually consistent with elements described in such models [69]. Future research incorporating validated behavioral measures would be needed to examine these theoretical relationships more rigorously.

### Practical implication

These findings provide insights that may inform antibiotic stewardship strategies in Damascus and similar settings (Table S11). The observed association between antibiotic knowledge and responsible practices (Table 2; Fig. 4) highlights opportunities for targeted public health interventions. The results highlight important gaps in public awareness of antibiotic ABR and appropriate antibiotic use. Participants with lower education levels showed a lack of awareness regarding ABR. Developing culturally appropriate, accessible awareness campaigns—leveraging local languages and communication channels such as social media, community centers, and schools—can substantially improve understanding of antibiotic indications, side effects, and resistance risks. This approach addresses the observed knowledge gaps and behavioral misconceptions, such as the unsafe reuse of antibiotics (Fig. 4), which pose direct risks to public health.

Moreover, the prominent role pharmacists play as accessible healthcare providers (correlated with advice-seeking behavior, *r* = 0.70, *p* < 0.01) suggests that integrating pharmacists more formally into antibiotic stewardship programs would yield immediate benefits. Strengthening pharmacists’ involvement in patient counseling may enhance community-level antibiotic stewardship. This strategy is particularly feasible in settings like Damascus, where physicians may be less accessible due to healthcare resource constraints.

The significant misuse of antibiotics in agriculture, reported by nearly 60% of farmers in Damascus, highlights the urgent need to extend stewardship beyond human medicine. Implementing regulatory frameworks that mandate veterinary oversight for antibiotic use in livestock, accompanied by farmer education on the environmental and public health impacts of misuse, is essential. Collaboration between agricultural agencies, public health authorities, and local communities can ensure these policies are practical and enforceable, mitigating AMR emergence from agricultural sources.

Additionally, the financial and insurance challenges faced by over 60% of respondents underscore that economic barriers limit access to professional healthcare consultation, associated with risks of AMR development. Policymakers should consider subsidizing healthcare visits or developing low-cost telemedicine platforms to facilitate timely medical advice, thereby reducing self-medication risks [70].

Finally, the documented knowledge-behavior link suggests that monitoring and evaluation frameworks must be incorporated into ongoing stewardship initiatives to assess knowledge acquisition and behavioral change over time. This evidence-based feedback will enable iterative program refinement, optimizing resource allocation and maximizing impact.

Overall, the findings support the value of coordinated interventions involving public education, healthcare professionals, and regulatory frameworks to improve antibiotic stewardship. These practical measures are both logically grounded in the presented data and feasibly adaptable within the socio-economic and cultural landscape of Damascus, providing a replicable blueprint for similar low- and middle-income regions facing AMR challenges (Table S11).

### Limitation and future study

A primary limitation of this study is the sample size (*n* = 200), which falls below the calculated requirement for high-precision generalizability. This smaller cohort increases the risk of Type II errors and may not capture the full diversity of the Syrian population. Consequently, the findings are preliminary and should serve as a pilot for larger, multi-city longitudinal studies. While this investigation provides valuable insights into patterns of antibiotic knowledge and self-reported behaviors among a sample of residents in Damascus, highlighting several associations between sociodemographic characteristics and antibiotic-related practices, several methodological limitations warrant consideration, including potential biases inherent to self-reported survey data. The study did not include microbiological sampling or laboratory-based measurement of AMR; therefore, the reported knowledge and behavioral patterns cannot be directly linked to actual antibiotic consumption patterns or to confirmed resistance outcomes within the study population. The supplementary tables summarizing antibiotic–pathogen relationships represent qualitative contextual information derived from established microbiological participant’s medical history files rather than primary resistance data from the study population [71]. Consequently, although the study identifies associations between antibiotic knowledge and self-reported behavioral practices, it cannot determine whether these behaviors correspond to actual antibiotic consumption patterns or translate into measurable AMR dynamics in the community.

Further, due to the cross-sectional design of this study, the observed relationships between variables are correlational rather than causal. We cannot determine whether increased knowledge preceded the adoption of responsible practices or if both are influenced by an external third factor, such as higher overall health literacy or education. Longitudinal studies are needed to track changes over time and better understand how educational interventions influence antibiotic use patterns. Likewise, this research relies on self-reported data obtained through structured questionnaires, which is inherently susceptible to several reporting biases, including social desirability bias and recall bias. Because antibiotic use behaviors were not verified through prescription records, pharmacy dispensing data, or clinical documentation, the study was unable to independently validate participants’ responses, and therefore the reported behaviors should be interpreted as perceived or reported practices rather than objectively measured antibiotic consumption patterns. Participants may therefore have over-reported socially desirable behaviors (such as adherence to prescribed antibiotic courses) or under-reported behaviors perceived as inappropriate (such as self-medication or sharing antibiotics), potentially affecting the accuracy of the behavioral estimates reported in this study. Additionally, because recruitment was conducted through face-to-face interviews in public spaces, the study may also be subject to selection bias, potentially favoring individuals who were more available or willing to engage with researchers during the sampling period; such contexts may further influence response behavior, as participants interviewed in public settings may feel inclined to provide socially acceptable answers. These factors, combined with the study’s restricted geographic scope within the urban context of Damascus, mean the findings should be generalized to the broader Syrian population with considerable caution, as antibiotic KP may differ substantially across rural areas, smaller cities, and regions affected by differing healthcare access and socioeconomic conditions. Hence, future research employing mixed methods—such as qualitative interviews and objective prescription audits—would enrich understanding of underlying motivations and real-world practices. Furthermore, the recruitment in public spaces may have introduced selection bias, potentially over-representing certain demographic groups (e.g., younger, more active individuals) and limiting the generalizability of the findings to the broader Damascus population. Future studies should expand the geographic scope to include multiple Syrian regions, particularly rural and peri-urban communities, and integrate objective data sources such as prescription records, pharmacy dispensing data, or microbiological surveillance systems to better link community behaviors with actual antibiotic consumption and resistance outcomes. Additionally, the investigation did not assess healthcare providers’ perspectives in depth, such as prescribing practices or continuing education uptake, which are pivotal components of antibiotic stewardship. Integrating healthcare professionals’ insights and system-level factors into future studies could illuminate barriers and facilitators within clinical contexts. In addition, although several behavioral and interdisciplinary frameworks (e.g., HBM, TPB, COM-B, and One Health perspectives) are referenced in the Discussion to provide conceptual context, the survey instrument did not include direct measures of the constructs underlying these models. Consequently, the study was not designed to test or validate these frameworks, and the theoretical interpretations should be viewed as exploratory rather than confirmatory. So, these frameworks are employed as post-hoc interpretive tools to contextualize the observed associations between knowledge and practice. Adds on, while the study touched upon agricultural antibiotic use, it lacked detailed exploration of environmental reservoirs and transmission pathways of resistance. Future multidisciplinary research incorporating environmental microbiology and veterinary sciences would align with a holistic One Health approach to AMR mitigation.

Finally, although trained interviewers followed standardized procedures and anonymity was emphasized to encourage honest responses, the possibility of reporting bias cannot be fully eliminated in survey-based KAP studies. Addressing all these limitations through longitudinal, mixed-methods, and multisectoral studies will provide deeper, more actionable insights and foster the development of targeted, sustainable interventions tailored to the complex socio-cultural landscape of antibiotic use.

#### Key research questions for future studies

- How do antibiotic knowledge and usage behaviors evolve longitudinally following targeted educational interventions in different demographic and geographic settings?
- What socio-cultural and economic factors may be related to antibiotic self-medication and adherence in rural versus urban communities?
- How do healthcare providers perceive barriers to antibiotic stewardship, and what training modalities are most effective in improving prescribing practices?
- What is the role of veterinary antibiotic use in local agricultural communities in shaping human ABR patterns?
- How does environmental contamination with antibiotics correlate with community-level resistance, and what mitigation strategies are most feasible?

## Conclusions

This study explored antibiotic-related knowledge and self-reported practices among a sample of Damascus residents and identified several associations between educational level, information sources, and antibiotic use behaviors. Improving public knowledge and responsible antibiotic practices is essential for mitigating antibiotic resistance (ABR) and contributing to broader efforts to address antimicrobial resistance (AMR).

While our findings contribute to the global effort to mitigate AMR, localized interventions in Syria must prioritize the reduction of ABR through targeted educational programs on rational antibiotic use. The data reveal a generally positive baseline of awareness regarding antibiotic use and AMR, yet significant misconceptions persist—particularly concerning self-medication and the reuse of leftover antibiotics—that pose tangible risks for accelerating resistance. Importantly, the suggested strong correlations between knowledge levels and responsible antibiotic behaviors underscore education as a cornerstone for effective antibiotic stewardship. These findings emphasize that improving antibiotic literacy must go beyond generic awareness campaigns; it requires targeted, evidence-based educational interventions that address specific misconceptions and behavioral drivers identified in this population. The significant influence of factors such as education level and healthcare consultation behaviors on antibiotic use further points to the need for integrated strategies involving healthcare providers, pharmacists, and community stakeholders. Moreover, the study sheds light on the critical role of pharmacists and the healthcare system as trusted sources of information, suggesting that empowering these actors are associated with responsible antibiotic practices. The insights gained provide a data-driven foundation for policymakers and health authorities to tailor public health programs that align with the socio-demographic realities of Damascus, ultimately contributing to the global effort against AMR. However, because the study design was cross-sectional, the relationships identified should be interpreted as associations rather than causal relationships.

In summary, this work highlights the indispensable role of targeted education, community engagement, and multi-sectoral collaboration in transforming antibiotic use patterns, thereby safeguarding the efficacy of antibiotics and enhancing population health resilience.

## Supplementary Information

Supplementary Material 1.

## Data Availability

The datasets used and/or analysed during the current study are available from the corresponding author on reasonable request.

## Abbreviations

ABR: Antibiotic Resistance
AMR: Antimicrobial Resistance
CI: Confidence Interval
COM-B: Capability, Opportunity, Motivation, and Behavior
HBM: Health Belief Model
KAP: Knowledge, Attitudes, and Practices
KP: Knowledge and Practice
OR: Odds ratio
TPB: Theory of Planned Behavior
