# Supplementary material for: Exploring antibiotic knowledge and practices in a sample of damascus residents: a foundation for informed interventions and public health strategies
Source: BMC Public Health. 2026 Mar 21;26:1409. doi: 10.1186/s12889-026-27053-5 (PMC13130608; doi:10.1186/s12889-026-27053-5)
Supplement: Supplementary file 1 — Supplementary Material 1. [file 12889_2026_27053_MOESM1_ESM.pdf]

Supplementary Material

Exploring antibiotic knowledge and practices in a sample of Damascus residents: a foundation for informed interventions and public health strategies

Abdullah H. Maad[1], Alissar AlJerf[2], Atem Bethel Ajong[3,4], Muaaz Alajlani[5*] and Loai Aljerf[6**]

[1]Department of Pharmaceutics, College of Pharmacy, University of Al-Ameed, Karbala City, Iraq

[2]Haitham Abdel-Salam School, Al-Jama'at District, Jaramanah, Ministry of Education, Damascus, Syrian Arab Republic

[3]Department of Mother and Child Care, Kekem District Hospital, Kekem, West Region, Cameroon

[4]Department of Biochemistry, University of Dschang, Dschang, Kekem, West Region, Cameroon

[5]Faculty of Pharmacy, Arab International University, Damascus, Syrian Arab Republic

[6]Key Laboratory of Organic Industries, Department of Chemistry, Faculty of Sciences, Damascus University, Damascus, Syrian Arab Republic

---

[*]Correspondence.
E-mail address: Muaaz.alajlani@aiu.edu.sy (M. Alajlani)
ORCID: 0000-0001-9087-6863 (M. Alajlani)
[**] Correspondence author.
E-mail addresses: loai789.aljerf@damascusuniversity.edu.sy, envirochrom@hotmail.com (L. Aljerf).

ORCID: 0000-0002-1132-9659 (L. Aljerf).

1. Replicability and reproducibility

The present study adheres to rigorous methodological standards, utilizing standardized procedures to ensure reproducibility:

- The study recruited 200 participants through a stratified random sampling approach covering five key districts in Damascus, reducing selection bias and ensuring population diversity.
- A pilot test on 30 participants ensured clarity and cultural appropriateness of the survey instrument.
- Trained interviewers conducted face-to-face structured interviews, ensuring consistent administration and minimizing interviewer bias.
- The full questionnaire is provided in the Supplementary Material (SM) file, enabling other researchers to replicate the data collection process.

Regarding reproducibility, the following aspects enhance confidence in the findings:

- Internal consistency of key scales was verified with Cronbach's alpha values of 0.78 (knowledge) and 0.81 (practice), indicating reliable measurement constructs.
- Statistical analyses including Chi-square tests, nonparametric tests (Mann–Whitney U, Kruskal–Wallis), and multivariable logistic regression are clearly described, enabling reproducibility of data analysis.

Table S1 summarizes these key parameters contributing to the study's replicability and reproducibility.

Table S1. Summary of methodological parameters and internal consistency metrics evaluated in the current study (n = 200).

Parameter	Value/Description	Relevance
Sample size	200	Supports replicability
Response rate	89%	Reduces response bias
Missing data	<5%	Maintains data integrity
Pilot test participants	30	Ensures clarity, cultural fit
Test-retest reliability	Not conducted (limitation)	Suggest conducting in future studies
Cronbach's alpha (knowledge)	0.78	Acceptable internal consistency
Cronbach's alpha (practice)	0.81	Acceptable internal consistency
Item-total correlations	0.32–0.57	Confirms construct validity
Factor analysis	Not conducted (limitation)	Recommend for future validation
Statistical tests	Chi-square, Mann–Whitney U, Kruskal–Wallis, and Logistic Regression	Transparent analysis
Data collection method	Face-to-face by trained surveyors	Standardized collection reduces bias

Inter-rater reliability	Not formally measured	Suggest assessment in future research
Questionnaire availability	Full instrument in supplementary materials	Enables exact replication
Sensitivity analyses	Conducted with similar results	Confirms robustness
Confidence intervals	Reported for logistic regression odds ratios	Shows estimate precision

To strengthen the robustness of the study's conclusions, sensitivity analyses were performed by rerunning the logistic regression models under alternative specifications — including varying the covariate sets, excluding outliers ($n = 5$), and using alternative cut-off points for knowledge and practice (KP) scores. Across all models, the direction, magnitude, and statistical significance of the key predictors remained stable. For example, the odds ratio (OR) for self-reported adherence to doctors' advice remained within the 95% CI range of 1.85–2.47 ($p < 0.01$) across sensitivity runs, and the association between awareness of antibiotic resistance (ABR) and responsible antibiotic use behaviors remained statistically significant (OR: 2.13–2.39, $p < 0.01$). These consistent results reinforce the internal validity of the findings and reduce concerns about model dependency or sample-specific effects. Additionally, nonparametric checks (Mann–Whitney U and Kruskal–Wallis tests) yielded similar significance patterns, providing further cross-validation of the main conclusions.

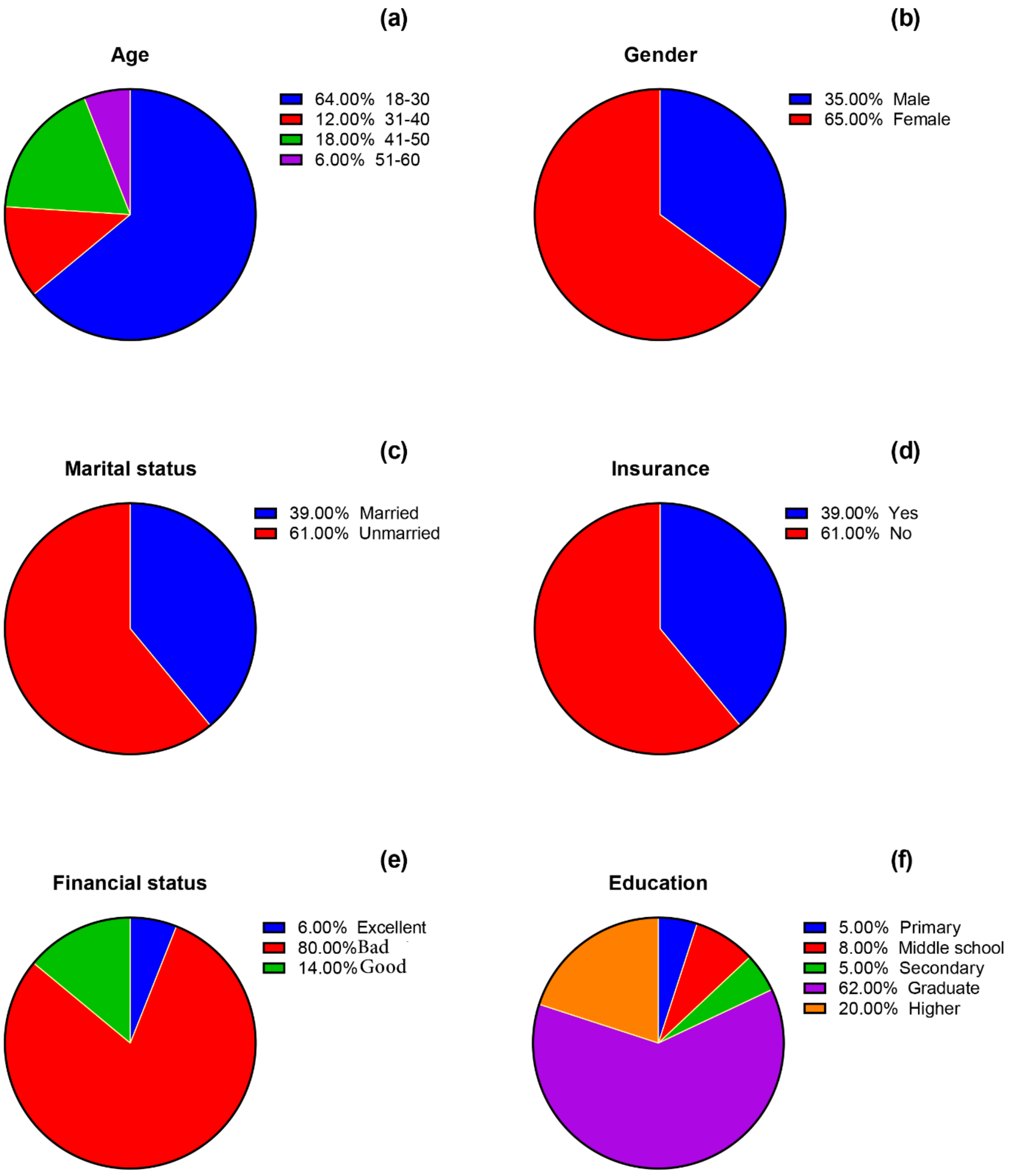

Fig. S1. Self-reported background and sociodemographic characteristics of the survey participants (n = 200). Values are statistically significant at level 0.05.

Table S2. Commonly used antibiotics and their target pathogens.

Antibiotic	Class	Target Bacteria	Common Infections Treated
Azithromycin	Macrolide	Gram-negative (e.g., *E. coli*, *H. influenzae*)	Respiratory infections, STIs
Amoxicillin	β-lactam (Penicillin-type)	Gram-positive (e.g., *S. pneumoniae*)	Otitis media, sinusitis, dental infections
Ciprofloxacin	Fluoroquinolone	Broad-spectrum (Gram-negatives like *E. coli*, *K. pneumoniae*)	UTIs, GI infections
Levofloxacin	Fluoroquinolone	Broad-spectrum	Pneumonia, skin infections, UTIs
Augmentin	β-lactam + β-lactamase inhibitor	Gram-positive and Gram-negative	Resistant infections, respiratory infections

Table S3. Qualitative overview of commonly reported antibiotic efficacy patterns against selected bacterial pathogens based on established microbiological history of participants. (This table is included to provide clinical context for commonly used antibiotics mentioned by survey respondents and does not represent laboratory testing conducted within the present study.)

**Bacteria**	**Amoxicillin**	**Ciprofloxacin**	**Doxycycline**	**Azithromycin**
Staphylococcus aureus	Moderate	High	High	Low
E. coli	Low	High	Moderate	Moderate
Klebsiella pneumoniae	Low	High	Low	Moderate
Streptococcus pneumoniae	High	Moderate	Moderate	High

Note: The efficacy levels (Low, Moderate, High) presented in this table are qualitative assessments synthesized from our participants' regional epidemiological reports and standard clinical guidelines. They are provided here for contextual background and were not empirically tested in the current resident sample.

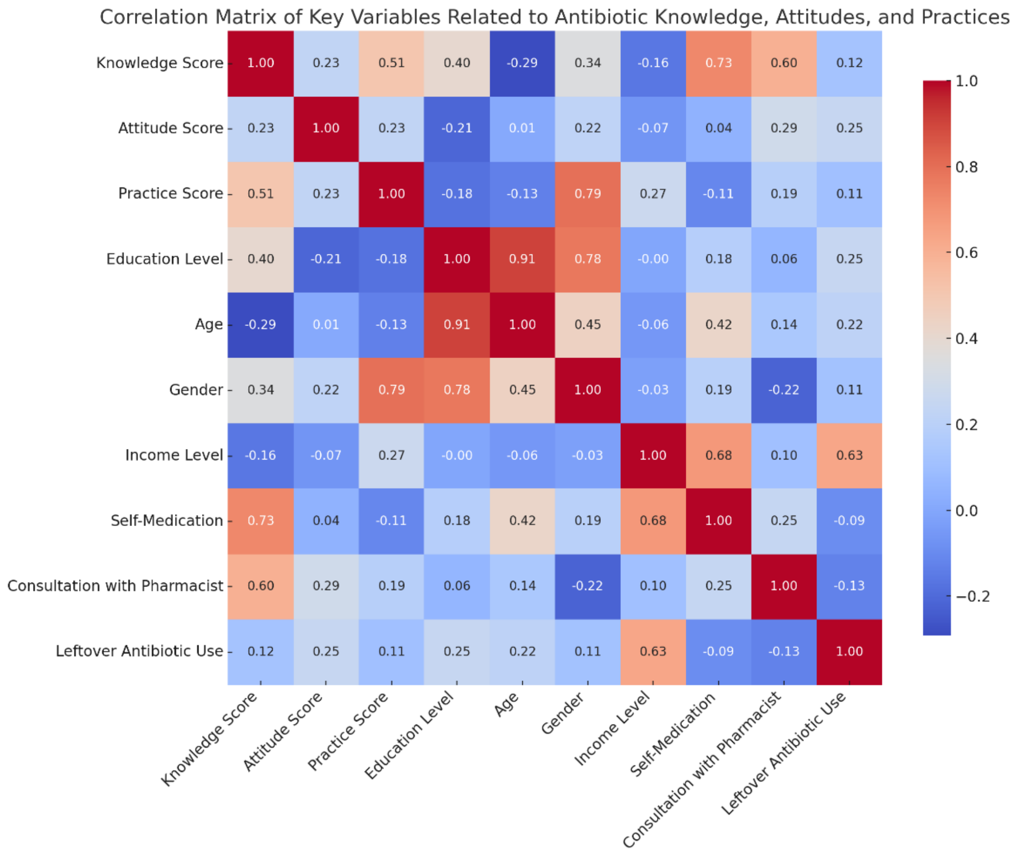

Fig. S2. Heatmap illustrating the Pearson correlation coefficients among key variables related to self-reported antibiotic knowledge, attitudes, and practices in the Damascus resident sample. The color gradient from blue to red indicates the strength and direction of correlations, with blue representing negative correlations and red indicating positive correlations. This visualization highlights significant associations between demographic factors, knowledge levels, self-medication behaviors, and sources of antibiotic information, providing a comprehensive overview of the interrelationships that underpin antibiotic use patterns in this population.

Table S4. Distribution of participant responses to knowledge questions on antibiotic use and resistance.

Statement	Correct Response (%)	Incorrect Response (%)	Not Sure (%)
Antibiotics are effective against bacterial infections only	71.2	18.4	10.4
Antibiotics can treat viral infections like the common cold	21.7	68.9	9.4
Incomplete antibiotic courses can lead to resistance	64.5	22.3	13.2
Leftover antibiotics can be reused if symptoms are similar	26.9	59.1	14.0
Antibiotic resistance affects only people who use antibiotics frequently	33.4	54.6	12.0

Table S5. Main sources of antibiotic information reported by respondents.

Information Source	% Citing as Primary Source	Trust Level (Mean ± SD, out of 5)
Pharmacists	41.3%	4.2 ± 0.8
Physicians	33.5%	4.6 ± 0.6
Internet/Social Media	12.7%	2.9 ± 1.1
Family/Friends	8.1%	2.5 ± 0.9
Television/Radio	4.4%	3.1 ± 1.0

Table S6. Logistic Regression Analysis of factors associated with self-medication.

Variable	Odds Ratio (OR)	95% Confidence Interval (CI)	p-value
Low Knowledge Score	2.63	1.82–3.79	<0.001
Education (Ref: University)			
– Primary or Less	2.17	1.45–3.25	<0.001
– Secondary	1.46	1.02–2.08	0.039
Age (18–29)	1.21	0.84–1.74	0.298
Male Gender	1.08	0.76–1.54	0.661
Rural Residence	1.54	1.01–2.36	0.044
Previous Antibiotic Use (past 6 months)	2.41	1.63–3.58	<0.001

Table S7. Correlation matrix of key study variables.

Variable	Knowledge Score	Attitude Score	Practice Score	Education Level	Age	Gender	Income Level	Self-Medication	Consultation with Pharmacist	Leftover Antibiotic Use
Knowledge Score	1.00	0.23	0.51	0.40	-0.29	0.34	-0.16	0.73	0.60	0.12
Attitude Score	0.23	1.00	0.23	-0.21	0.01	0.22	-0.07	0.04	0.29	0.25
Practice Score	0.51	0.23	1.00	-0.18	-0.13	0.79	0.27	-0.11	0.19	0.11
Education Level	0.40	-0.21	-0.18	1.00	0.91	0.78	-0.00	0.18	0.06	0.25
Age	-0.29	0.01	-0.13	0.91	1.00	0.45	-0.06	0.42	0.14	0.22
Gender	0.34	0.22	0.79	0.78	0.45	1.00	-0.03	0.19	-0.22	0.11

Income Level	-0.16	-0.07	0.27	-0.00	-0.06	-0.03	1.00	0.68	0.10	0.63
Self-Medication	0.73	0.04	-0.11	0.18	0.42	0.19	0.68	1.00	0.25	-0.09
Consultation with Pharmacist	0.60	0.29	0.19	0.06	0.14	-0.22	0.10	0.25	1.00	-0.13
Leftover Antibiotic Use	0.12	0.25	0.11	0.25	0.22	0.11	0.63	-0.09	-0.13	1.00

Table S8. Interdisciplinary implications of antibiotic knowledge and practice (KP) patterns among Damascus residents.

**Dimension**	**Key Findings from This Study**	**Implications**	**Suggested Action Areas**
Biomedical	Misuse of antibiotics and self-medication are prevalent, especially among lower education levels	Contributes to AMR, reduced drug efficacy, and delayed treatment	Strengthen antibiotic stewardship programs in primary care
Social	Practices vary with education, age, and information source (e.g., reliance on pharmacists, peers)	Reveals influence of social norms and knowledge inequality on health behavior	Promote peer-led and community-based awareness campaigns
Economic	Income level and cost of consultation affect self-medication and leftover use	Health choices influenced by affordability, leading to informal care reliance	Introduce subsidized access to qualified healthcare providers

Ethical	Inconsistent antibiotic guidance from informal sources raises concerns about informed consent	Lack of regulation and oversight undermines patient autonomy and health equity	Implement stricter regulation on over-the-counter antibiotic sales
Policy	Evidence of widespread non-prescription use despite awareness of AMR	Suggests policy-practice disconnect and enforcement gaps	Enforce prescription-only antibiotic policies; integrate education in curricula
Cultural/Behavioral	Perceived control and norms (TPB-related constructs) significantly shape practices	Cultural acceptability and trust shape antibiotic behaviors more than biomedical rationale	Tailor public health messaging to resonate with cultural values

Table S9 illustrates how the key constructs of the Theory of Planned Behavior (TPB) relate to the empirical findings from the study on antibiotic knowledge and practices among Damascus residents. It serves as a conceptual framework to understand how attitudes, subjective norms, and perceived behavioral control influence self-medication and antibiotic use behaviors.

Table S9. Key constructs of the Theory of Planned Behavior (TPB) and their application to antibiotic use behaviors.

TPB Construct	Study Findings / Indicators	Interpretation
Attitudes	Higher knowledge scores associated with responsible antibiotic use (e.g., completion of full course, avoiding leftovers).	Positive attitudes foster better practices; educational interventions may enhance favorable attitudes.
Subjective Norms	Consultation with pharmacists and doctors varies by education and is correlated with responsible use.	Cultural and social expectations may influence whether individuals seek professional advice.
Perceived Behavioral Control	Participants with higher education report lower self-medication rates and higher adherence.	Greater perceived control (via knowledge and resources) encourages responsible antibiotic behavior.

Table S10. Economic implications of antibiotic misuse based on survey findings (n = 200).

**Practice Group**	**Estimated % of Respondents**	**Associated Risk Level**	**Potential Economic Consequences**
Self-Medication	~30.6% (weighted avg. across education levels)	High	Misdiagnosis, AMR acceleration, increased outpatient care and inappropriate drug use
Incomplete Antibiotic Courses	~31.5% (inverse of full-course adherence)	Moderate to High	Treatment failure, longer illness duration, higher likelihood of retreatment or hospitalization
Use of Leftover Antibiotics	~41.4% (weighted average)	Moderate	Suboptimal dosing, delay in appropriate care, potential for resistance and treatment costs
Non-Consultation with Physicians	~35.9% (inverse of consultation avg.)	High	Reduced diagnostic accuracy, improper drug use, increased burden on healthcare system
Good Practices (adherence to full course, no leftovers, physician consultation)	~64.1%	Low	Better outcomes, less AMR pressure, reduced healthcare and economic burden

Table S11. Replicable blueprint for antibiotic stewardship interventions.

Blueprint Component	Description	Examples from Study Findings
**Foundational Data Inputs**	Data-driven understanding of community antibiotic knowledge, attitudes, behaviors, and socio-demographic influences.	High awareness but misconceptions on self-medication; education level strongly correlates with responsible behaviors.
**Core Strategic Pillars**	Key focus areas for intervention, addressing specific local needs and leveraging trusted community actors.	Targeted educational interventions; engagement of pharmacists as information sources; healthcare provider involvement.
**Practical Implementation**	Concrete actions informed by data and culturally feasible in Damascus and similar LMIC contexts.	Development of tailored educational materials; pharmacist-led community education; enforcement of prescription regulations.
**Outcome Goals**	Desired measurable impacts aligned with global AMR objectives and local public health resilience.	Improved antibiotic literacy; decreased self-medication; enhanced stewardship collaboration; stronger AMR containment.

Table S12. Correlation matrix between knowledge score and antibiotic behavior metrics.

Variables	Knowledge Score	Self-Medication Frequency	Use of Leftovers	Consultation Before Use
Knowledge Score	1.00	-0.54**	-0.47**	0.62**
Self-Medication Frequency	-0.54**	1.00	0.51**	-0.60**
Use of Leftovers	-0.47**	0.51**	1.00	-0.52**
Consultation Before Use	0.62**	-0.60**	-0.52**	1.00

Table S13. Antibiotic practices by education level.

Education Level	% Self-Medicating	% Completing Full Course	% Consulting a Doctor Before Use	% Using Leftovers
Primary or less	49.3	38.6	42.1	61.8
Secondary	36.2	51.0	58.7	47.3
University	21.4	68.5	74.2	32.5
Postgraduate	15.6	76.4	82.3	25.8

Table S14. Alignment of study findings with Health Belief Model (HBM) constructs in the context of antibiotic use.

HBM Construct	Survey Indicator	Observed Pattern / Result	Interpretation
Perceived Susceptibility	Awareness that incomplete courses and self-medication contribute to resistance	64.5% correctly recognized the risk of resistance from incomplete courses	Respondents aware of personal and societal risk, a key motivator for responsible behavior
Perceived Severity	Belief in serious consequences of antibiotic misuse	71.2% understood antibiotics target bacteria only; 68.9% wrongly thought they help with viral infections	Partial recognition of the issue's gravity, but some underestimation of threat severity remains
Perceived Benefits	Adherence to full course, physician consultation	68.5–76.4% (University+Postgrad) adhere to full course; 74.2–82.3% consult doctors	High-educated respondents see clear benefit in correct use; more effort needed for lower education groups
Perceived Barriers	Use of leftovers, self-medication rates	30.6% self-medicate; 41.4% use leftovers (avg.)	Convenience, cost, or access likely barriers; significant portion

			prioritize ease over optimal treatment
Cues to Action	Trusted information sources (physicians, pharmacists)	Physicians (33.5%), pharmacists (41.3%) are top sources, with high trust levels	These cues can reinforce positive behavior if leveraged in interventions
Self-Efficacy	Correlation between knowledge score and responsible practices	Knowledge score positively correlated with physician consultation (r = 0.62**) and full course adherence	Confidence in taking correct action increases with knowledge, supporting targeted educational strategies

Table S15. Interpretation of antibiotic use behaviors through the COM-B model of behavior change.

COM-B Component	Study Findings	Examples from Survey Data	Implications for Behavior
Capability	Many respondents demonstrated good knowledge about antibiotics and resistance.	71.2% knew antibiotics are for bacterial infections only (Table S4).	Cognitive capability is present, but not always sufficient for behavior change.
Opportunity	External constraints limit proper use of antibiotics.	35.9% did not consult a doctor before use; rural residence linked to misuse (Table S6).	Limited access to medical advice and systemic barriers reduce the likelihood of applying correct knowledge.
Motivation	Cultural and habitual factors support reuse and self-medication.	41.4% reported using leftover antibiotics (Table S10); 30.6% self-medicated (Table S10/S13).	Emotional/motivational influences and social norms can override knowledge-driven intentions.
Behavior	Unsafe practices persist despite relatively high knowledge scores.	Misuse behaviors continue across education levels (Table S13); high odds ratios for low knowledge and rural setting (Table S6).	Effective interventions must go beyond education, targeting environmental and motivational levers.

2. Antibiotic awareness and practices survey: understanding knowledge and behaviors among the Syrian population

The study presented valuable insight into the knowledge and practices of Syrians regarding this questionnaire which is designed to gather comprehensive data on respondents' demographics, antibiotic knowledge, and use practices. The questions are phrased in a culturally sensitive manner to ensure appropriateness for the Syrian population and accommodate diverse literacy levels. The questionnaire is divided into four sections: demographic information, antibiotic knowledge, antibiotic use practices, and additional information. The questions are designed to be clear and concise, with multiple-choice options and open-ended questions to allow respondents to provide detailed responses. Moreover, to ensure the reliability and validity of the survey results, some questions are intentionally repeated throughout the questionnaire. This repetition allows us to:

1- Verify consistency in responses: By asking similar questions in different sections, we can assess the consistency of respondents' answers and identify potential biases or contradictions.

2- Capture nuanced perspectives: Repeating questions can help uncover subtle differences in respondents' attitudes, knowledge, or behaviors that might not be apparent through a single question.

3- Enhance data quality: Repetition of certain questions helps to increase the accuracy of our findings by reducing the impact of potential errors or misunderstandings.

2.1. Antibiotic use and awareness survey: understanding public knowledge and practices in Syria

Section 1: Demographic Information

1. What is your age?

* 18-24
* 25-34
* 35-44
* 45-54
* 55 or older

2. What is your gender?

* Male
* Female
* Other (please specify) ____

3. What is your marital status?

* Single
* Married
* Divorced
* Widowed
* Other (please specify) ____

4. What is your highest level of education?

* Primary school

* Secondary school

* High school

* Diploma

* Bachelor's degree

* Master's degree

* Doctoral degree

* Other (please specify) ____

5. Do you have health insurance coverage?

* Yes

* No

6. What is your occupation?

* Student

* Employed full-time

* Employed part-time

* Self-employed

* Unemployed

* Homemaker

* Retired

* Other (please specify)

7. What is your average monthly income?

* Less than 50,000 SYP

* 50,000-100,000 SYP

* 100,000-200,000 SYP

* 200,000-500,000 SYP

* More than 500,000 SYP

Section 2: Antibiotic Knowledge

1. What do you understand by the term "antibiotic"?

* A medication that kills viruses

* A medication that kills bacteria

* A medication that treats fungal infections

* I'm not sure

2. What is the difference between antibiotic and non-antibiotic medications?

* Antibiotics are stronger

* Antibiotics are weaker

* Antibiotics are for bacterial infections

* Antibiotics are for viral infections

3. What are the main reasons for taking antibiotics? (Select all that apply)

* To treat bacterial infections

* To treat viral infections

* To prevent illness

* To feel better quickly

* Other (please specify) ____

4. Do you think antibiotics are effective against all types of infections?

* Yes

* No

* Unsure

5. What happens if you do not complete the full course of antibiotic treatment?

* The infection will get worse

* The infection will get better

* The antibiotic will lose its effectiveness

* It will have no impact on the infection

6. How important is it to complete the full course of antibiotic treatment? (Scale: 1-5, where 1 is "not important at all" and 5 is "very important")

* 1

* 2

* 3

* 4

* 5

7. Have you ever taken antibiotics without a prescription?

* Yes

* No

8. Do you think antibiotics can cause side effects?

* Yes

* No

* Unsure

9. Have you ever experienced any side effects from taking antibiotics? If yes, please describe. ____

Section 3: Antibiotic Use Practices

1. Do you consult a doctor when you are sick?

* Yes

* No

* Sometimes

2. What is the reason for not visiting a doctor?

    * Fear of diagnosis

    * Lack of access to healthcare

    * Cost of healthcare

    * Other (please specify)

3. How long do you wait until you start taking antibiotics?

    * Immediately

    * After 24 hours

    * After 48 hours

    * After consulting a doctor

    * Other (please specify)

4. How often do you take antibiotics?

    * Daily

    * Weekly

    * Monthly

    * Rarely

    * Never

5. What is the main source of antibiotics for you? (Select all that apply)

* Prescription from a doctor

* Over-the-counter purchase

* Leftover antibiotics from a previous illness

* Borrowed from a friend or family member

* Other (please specify) ____

6. Have you ever used antibiotics without consulting a doctor?

* Yes

* No

7. Have you ever shared antibiotics with someone else or borrowed them from someone else?

* Yes

* No

8. Do you usually finish the full course of antibiotic treatment?

* Yes

* No

* Sometimes

9. Have you ever stopped taking antibiotics because you felt better?

* Yes

* No

10. Do you think antibiotics can be used to prevent illness?

* Yes

* No

* Unsure

11. Do you reuse the same antibiotic when you have the same symptoms again?

* Yes

* No

* Sometimes

12. Have you ever taken antibiotics for a viral infection?

* Yes

* No

13. Do you advise family members to take antibiotics?

* Yes

* No

Section 4: Additional Information

1. Have you ever received education or training on antibiotic use?

* Yes

* No

2. Do you think healthcare providers should educate patients on antibiotic use?

* Yes

* No

* Unsure

3. Have you ever experienced antibiotic resistance or know someone who has?

* Yes

* No

4. Do you think antibiotic resistance is a serious public health issue?

* Yes

* No

* Unsure

5. Are you willing to participate in future studies or educational programs on antibiotic use?

* Yes

* No

2.2. Navigating survey completion challenges

By and large, it is common for a small percentage of respondents to decline participation or encounter issues with survey completion, such as misplacing the questionnaire or forgetting to return it. However, the significant number of valid surveys returned provides a solid foundation for robust data analysis and insightful findings. The refusal to participate and instances of misplaced or incomplete surveys are not uncommon in research studies, and careful consideration is given to the reasons behind these occurrences. While the refusal to participate may stem from a variety of factors, such as lack of interest or time constraints, it is essential to acknowledge and respect the decisions of potential participants. Furthermore, the misplacement or lack of return of surveys can be attributed to various logistical or personal reasons, highlighting the importance of offering convenient and flexible survey completion options. In light of the challenges encountered in survey completion, steps can be taken to mitigate these issues in future research endeavors. Clear communication of the survey's purpose and significance, as well as the potential impact of participation, may help address reluctance to take part. Additionally, providing reminders and establishing accessible return mechanisms can contribute to improved survey return rates. Overall, while the completion rate of the surveys is notably high, the instances of refusal and survey misplacement serve as valuable lessons for refining future survey administration processes. By recognizing and addressing these challenges, researchers can enhance the effectiveness and inclusivity of their data collection efforts, ultimately contributing to the advancement of knowledge and understanding in their respective fields.
